# “I Can't Get No Satisfaction”: Measuring Student Satisfaction in the Age of a Consumerist Higher Education

**DOI:** 10.3389/fpsyg.2017.00980

**Published:** 2017-06-08

**Authors:** Carl Senior, Elisabeth Moores, Adrian P. Burgess

**Affiliations:** Department of Psychology, School of Life and Health Sciences, Aston UniversityBirmingham, United Kingdom

**Keywords:** student engagement, student satisfaction, data primacy, measurements, innovation, universities

One could be excused for failing to recognize today's universities as the inheritors of the global higher education system that arose more than 70 years ago from the ashes of the Second World War. A wave of post-war optimism ushered in a global movement with a utopian vision in which arbitrary divisions such as class, gender, and race would be transcended in the pursuit of academic enlightenment (Scott, 1995). Universities were to be one of the key drivers of this change. But, contemporary academia is a distinctly different beast. The enlightenment values of the liberal education model, once the dominant philosophy in universities across the world, are gradually being supplanted by a consumerist ideology (Furedi, 2011): Yesterday's “*Cathedrals of learning*” are being replaced by today's “*Supermarkets of facts*^1^”.

The rise of the consumer model of universities, derided by many, has brought distinct benefits that the enlightenment model failed to achieve. One could perhaps marvel at the fact that here is a single philosophy that has effectively transcended national boundaries. By advocating a consumerist philosophy, managers of Higher Education (HE) institutions have been able to employ the full gamut of market forces to drive innovation in their day-to-day practice (Christensen and Eyring, 2011). Not least of the achievements arising from this, has been the massive expansion of the franchise such that university education, once the prerogative of a small social elite who valued learning for the sake of enlightenment, is now the expectation of a large proportion of the population whose primary desire is to improve their position on the subsequent employment market (Tomlinson, 2008). Today's universities have been quick to meet this need and institutional offerings have followed suit, enabling students to gain experience in a range of additional and subsidiary programmes that focus on the provision of “value added” benefits (Deane and Stanley, 2015). Here, students are encouraged to develop a wide range of transferable skills from entrepreneurship and enterprise to a knowledge of intellectual property rights and even leadership skills.

The embrace of the Business-to-Consumer model of HE also presents university managers with many challenges (See e.g., Deloitte's, 2015; “Making the grade” report). What does it mean to be a university in the modern consumerist era? How can the traditional values of scholarship and standards be preserved in a customer-focussed institution? How does the HE sector continue to enable graduates to become effective citizens who contribute to the betterment of society? Most important of all from the consumer model perspective, “What do students actually expect from HE and how are education providers framing and meeting these expectations?” The key metric for this last question is student satisfaction, yet, despite its almost ubiquitous position as a tool for university managers, the concept of “student satisfaction” remains ephemeral and surprisingly little is known about what makes a student satisfied with their experience of HE or how it can be measured effectively.

Only in the last 10 years or so has work emerged that has started to examine the institutional drivers of student satisfaction (Mai, 2005). Clemes and colleagues examined the various relationships between a range of institutional factors and their relationship to satisfaction in the student cohort (Clemes et al., 2008). They found a significant relationship between satisfied students and the quality of the teaching with a mediating role for institutional reputation. A significant predictive relationship was also reported between satisfaction and intended future outcomes post-graduation. Alves and Raposo (2007) also examined the behaviors that effectively predicted student satisfaction and also revealed that the quality of teaching experience was a key driver. More surprisingly, they also found that institutional reputation was actually a more influential predictor of student satisfaction than teaching quality. So, it would seem that students are satisfied if they receive good teaching at a reputable institute.

Alves and Raposo (2007) went on to examine the effects of having a cohort of satisfied students. They found that satisfaction bred loyalty. Students who were satisfied were more loyal to the institution and were more likely to engage with alumni activities and maintain an ongoing relationship with their alma mater. As universities in many countries expend considerable effort and money on establishing a body of loyal graduates that may one day reward them with a financial return, this is clearly an important finding. Gibbons et al. (2015) show that NSS scores have a small but statistically significant effect on University applications at a subject level, but suggest that this effect is primarily driven by league table positions (rather than original data).

The measurement of student satisfaction is one that undoubtedly vexes institutional managers around the world because, despite its importance, measuring satisfaction is not trivial and presents a number of challenges (see e.g., Cashin, 1990). For example, how can the new and emerging expectations of students be measured in an effective fashion? How can data be collected in a timely manner to ensure that managers can effect improvement in the immediate learning environment? How can we encourage the free flow of information from the consumers to the managers and vice versa that is so important to maintaining success in the modern competitive HE environment? Within HE, managers collect information on student satisfaction using a range of mechanisms designed to ensure that the expectations of the student are met at every stage of their progression through university. Timetabled one-to-one meetings between staff and students, drop-in sessions and staff-student consultative committees are now so pervasive that only the most insulated of academics can have failed to recognize the changing zeitgeist. Although these devices may be effective at the individual level, these strategies probably have little impact at the institutional level and almost none across the sector as a whole.

To address this problem, most developed countries use some form of national survey that they deliver to students to collect a range of measures of student satisfaction. Japanese academic managers make use of results from the Japanese College Student Survey (JCSS) and the Japanese Freshman Survey (JFS) both of which have been studied extensively (see e.g., Yamada, 2013). The National Survey for Student Engagement (NSSE) is used in the USA (Kuh, 2003). The Course Experience Questionnaire (CEQ) is employed in Australia (Ramsden, 1991; Wilson et al., 1997) and in the UK the National Student Survey (NSS: Richardson et al., 2007) is completed by almost 300,000 final year undergraduate students each year. In the UK, national league tables of the NSS results are published annually and are readily available to anyone contemplating applying to university. As such, NSS scores are an important driver of institutional change and woe betide the subject group or individual teacher who is perceived to be adversely affecting student ratings. Despite its influence, however, there is considerable debate as to whether the NSS offers sufficient discrimination between Universities to be useful, or measures fairly across different subject disciplines (Cheng and Marsh, 2010; Yorke et al., 2014).

The one consistent finding of all this work is that high quality teaching is an important factor in student satisfaction; a finding that should surprise no one. Excellence in teaching is the *sine qua non* of a modern university and the power of consumer choice alone is enough to ensure that a university which does not deliver its key product (effective teaching) to its consumer base (students) does not remain in business (see e.g., Mathooko and Ogutu, 2015; Milian et al., 2016). But why then is so much effort and cost^2^ dedicated to measuring aspects of student attitudes when the results are so clearly aligned with common sense?

One reason may be that the role of universities is changing. The rise of wide scale reforms across the global HE sector are inexorably driving University management away from the delivery of effective teaching toward the delivery of a more transferable and professional skillset that is more closely aligned to the graduate expectations of successful employment. On first consideration such a development may seem at odds with the traditional and clearly non-vocational model of a university which first emerged in the mid-nineteenth century with the early writings of Cardinal John Henry Newman^3^. Yet, even within this early philosophy there existed a clear advocacy for the development of skills acquired through general critical and reflective abilities that were applicable to any role in the workplace. In this model, university learning was less about employment and more about the ability to be successful in society, whereas in contemporary HE the development of a focused professional skillset has become an increasingly dominating influence. Indeed, in our view, it is likely that today's satisfied students are most likely to be those who have experienced a programme of study that aligns itself directly with their expectations for subsequent and very specific employment.

Such a shift is inevitable and, as we have previously argued, in order to deliver an effective learning experience the modern day university manager needs to embrace the full scope of the student activities that occur both on and off campus (Senior et al., 2014). This portfolio of experience should include the development of professional skills that they have acquired outside the classroom and in the world of work. However, as noted above this can be a vast and wide portfolio of professional skills (see also Bridges, 1993; Moores and Reddy, 2012; Reddy and Moores, 2012). Whilst institutions across the global HE sector are readily aligning the student experiences within the campus to meet these external expectations and deliver a truly engaged model of scholarship, they tend to lack the means to measure these activities and to ensure that modern day students are satisfied with the learning experience they receive (Van de Ven, 2007).

Upon reflection, we now make three recommendations for institutional managers and policy directors to consider. First, the academic environment has changed; managers can no longer expect students to be satisfied with excellent teaching alone. Students expect the provision of excellence with regards to professional skills that they can transfer to the post-graduation workforce and thereby harvest the economic and social benefits that attracted them to University study in the first place. Second, there needs to be a detailed and thorough statistical examination of the current means by which student satisfaction is measured across the HE sector. In our view, current measures of student satisfaction are no longer adequate in scope to meet the changing needs of students and the developing roles of universities. Third, and perhaps most important, there is a need to better understand the concept of student satisfaction and how this is driven by the increasingly important economic consequences that studying in HE has for individual students. In short, student satisfaction is a key concept in the modern consumerist HE sector, but it is one that we still don't fully understand and don't know how to measure.

## Author contributions

All authors listed have made substantial, direct and intellectual contributions to the work, and approved it for publication.

### Conflict of interest statement

The authors declare that the research was conducted in the absence of any commercial or financial relationships that could be construed as a potential conflict of interest.

## Footnotes

^1^Phrase attributed to the late Dr. Mike Harris of the University of Birmingham, UK.

^2^In the UK student satisfaction is measured with the annual National student Survey (NSS) that costs £2.4M to develop and deliver. Source: http://webarchive.nationalarchives.gov.uk/20120118164922/http://hefce.ac.uk/pubs/board/2004/93/B39.pdf

^3^John Henry Newman, in his seminal essay “*The Idea of a University” (1852)* made a powerful and influential case for the liberal ideal of a university.
